# A Rare Case of Pancreatic Cancer: Undifferentiated Carcinoma of the Pancreas With Osteoclast-Like Giant Cells

**DOI:** 10.7759/cureus.25118

**Published:** 2022-05-18

**Authors:** Ammar Ashfaq, Nishanth Thalambedu, Muhammad Umair Atiq

**Affiliations:** 1 Internal Medicine, Jefferson Health Abington, Abington, USA; 2 Internal Medicine, University of Arkansas for Medical Sciences, Little Rock, USA; 3 Medical Oncology, Jefferson Health Abington, Abington, USA

**Keywords:** osteoclast-like giant cells, pancreatitis, giant cancer, oncology, pancreatic cancer

## Abstract

Ductal adenocarcinoma of the pancreas is the most common pancreatic cancer, but undifferentiated carcinoma of the pancreas with osteoclast-like giant cells (UC-OGCs) is an exceedingly rare tumor. Microscopically, this tumor is characterized by the presence of two different cellular elements, namely, spindle or ovoid mononuclear cells and osteoclast-like giant cells (OGCs). Here, we report a rare case of UC-OGCs in a 79-year-old male with a one-month history of epigastric abdominal pain and unintentional weight loss. A blood workup revealed new-onset type 2 diabetes mellitus, and a computed tomography scan of the abdomen showed acute pancreatitis with a hypodense lesion in the head of the pancreas concerning for malignancy. He underwent an endoscopic ultrasound that also revealed a mass in the head of the pancreas, but no lymphadenopathy was observed. Biopsy was obtained and histopathology revealed UC-OGCs. We present this case to increase awareness of this rare clinical entity in patients presenting with acute-onset pancreatitis.

## Introduction

Pancreatic cancer has emerged as the seventh most common cause of cancer-related death worldwide. Even though pancreatic ductal adenocarcinoma is the most common pancreatic cancer, undifferentiated carcinoma of the pancreas with osteoclast-like giant cells (UC-OGCs) is an exceedingly rare exocrine tumor, accounting for less than 1% of all pancreatic malignancies [1]. Microscopically, this tumor is characterized by the presence of two different cellular elements, namely, spindle or ovoid mononuclear cells and osteoclast-like giant cells (OGCs) [2].

## Case presentation

**Figure 1:**
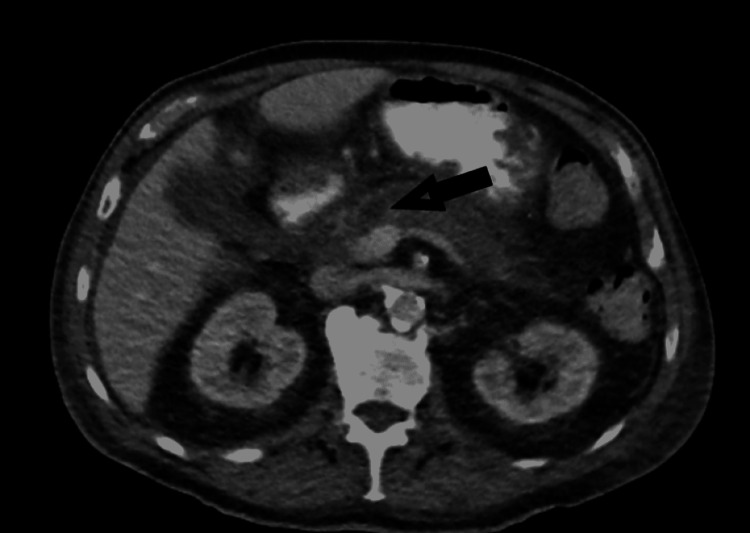
Hypodense lesion in the head of the pancreas.

A 79-year-old male initially presented to his primary care provider for evaluation of month-long epigastric pain and unintentional weight loss of 10 lbs. His medical history was significant for coronary artery disease, hypertension, and atrial fibrillation. His vital signs were not significant except for an irregularly irregular pulse. Physical examination was normal except for epigastric tenderness. His blood work showed glycated hemoglobin (HbA1C) of 8.3% (normal: 5.7-6.4%), blood glucose of 383 mg/dL (normal: 40 mg/dL or below), bilirubin of 1.2 mg/dL (normal: 0.3-1.2 mg/dL), aspartate aminotransferase (AST) of 25 U/L (normal: 0-35 U/L), alanine aminotransferase (ALT) of 19 U/L (normal: 0-35 units/L), lipase of 271 U/L (normal: 0-95 U/L), amylase of 100 U/L (range: 0-130 U/L), blood urea nitrogen (BUN) of 34 mg/dL (normal: 8-20 mg/dL), creatinine of 1.6 mg/dL (normal: 0.7-1.3 mg/dL), white blood cell count (WBC) of 7.9 k/µL (normal: 4,000-10,000/µL), hemoglobin of 10.5 g/dL (normal: 14.0-17.0 g/dL), and platelet count of 169,000 k/µL (normal: 150,000-350,000/µL). Cancer antigen 19-9 was 534 U/mL (normal: 0-37 U/mL). The patient underwent computed tomography (CT) scan of the abdomen and pelvis with intravenous and oral contrast which was suggestive of a 2 cm hypodense lesion in the head of the pancreas (Figure 1). Given these findings, a plan was made to proceed with endoscopic ultrasonography (EUS) with biopsy which revealed a complex cystic mass in the head of the pancreas with infiltration into the second part of the duodenum. Biopsies from the cystic mass were obtained.

**Figure 2:**
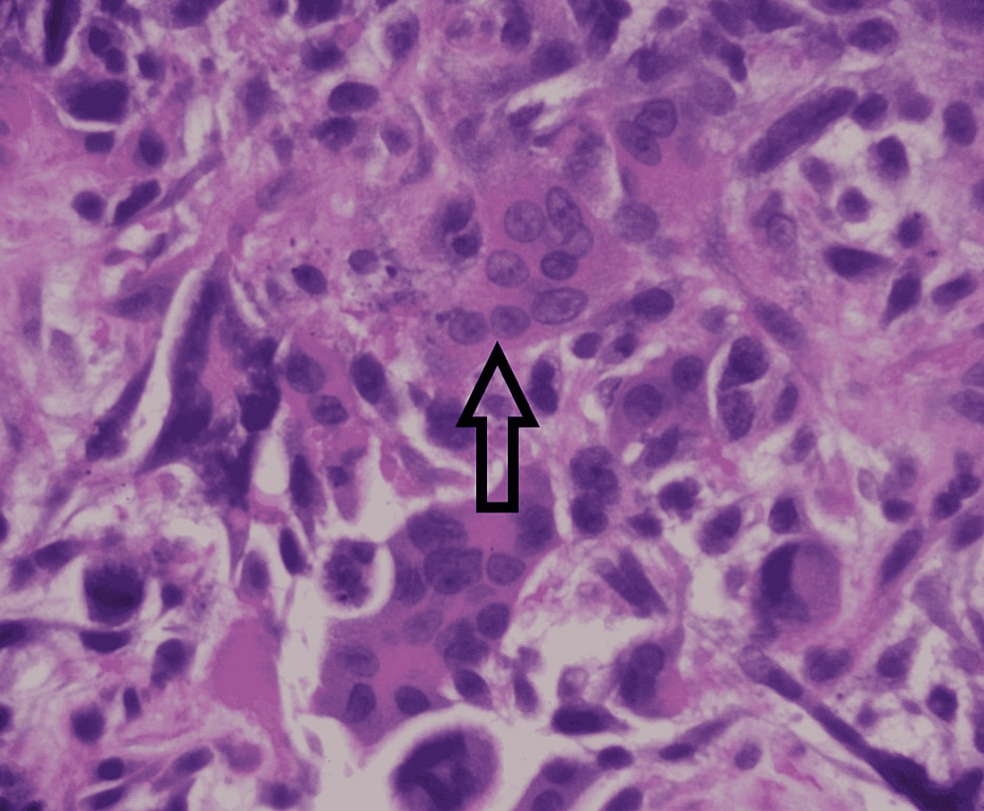
Sarcomatous-appearing tumor with multinucleated giant cells.

**Figure 3:**
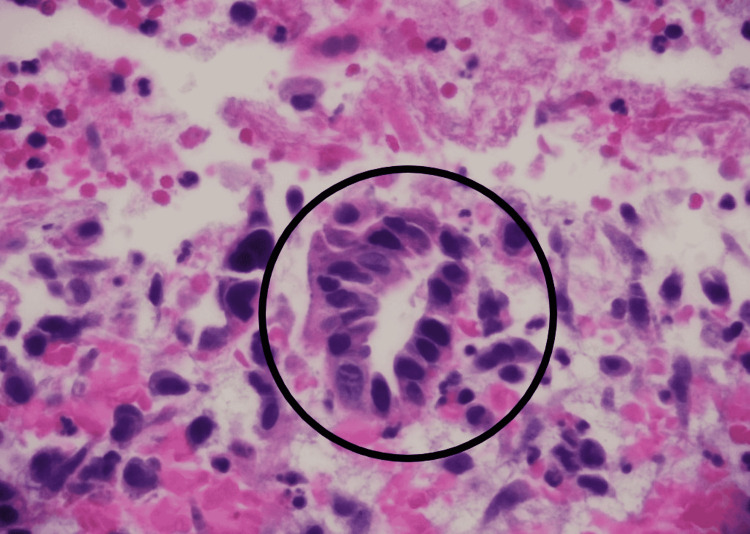
Malignant gland-like structure.

**Figure 4:**
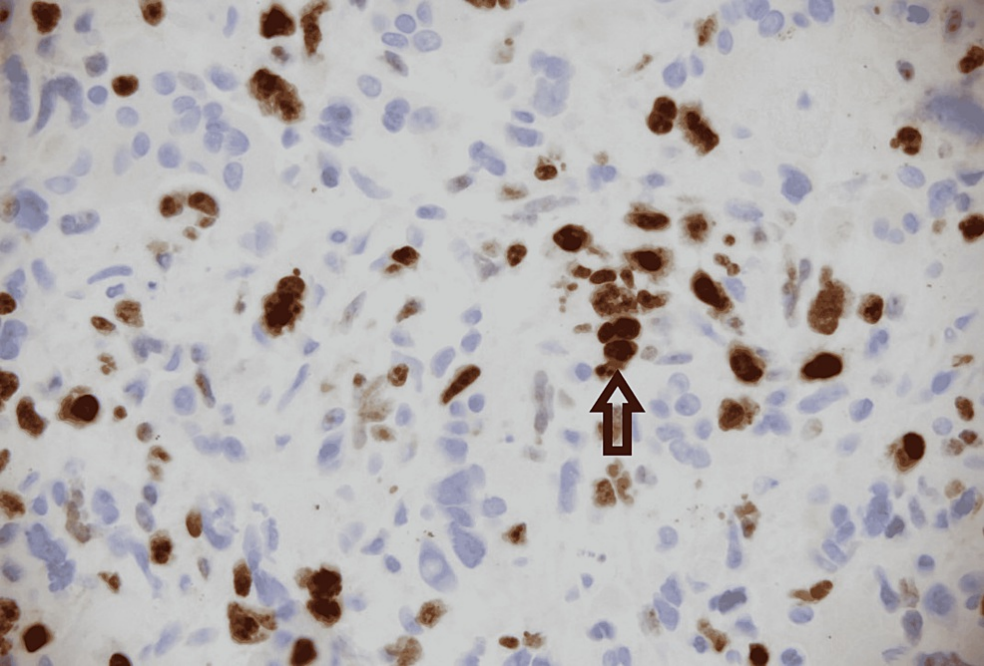
An immunohistochemical stain for Ki67.

Microscopy was suggestive of groups of abnormal spindled and epithelioid cells intermixed with numerous multinucleated giant cells. Some of the giant cells appeared bland (osteoclast-like) (Figure 2), and others exhibited highly pleomorphic, bizarre nuclei. Rare abnormal gland-like formations and focal necrosis were also seen (Figure 3). On immunohistochemical stains, most lesion cells were strongly positive for vimentin. Pan-cytokeratin stain also showed the rare glandular elements as well as faint focal staining of the spindled tumor cells. Stain for CD68 highlighted the giant cells and the intermixed population of histiocyte-like sarcomatous carcinoma cells. Among the non-giant cell population, Ki67 stain demonstrated a proliferation index of approximately 30% (Figure 4). All these findings were typical for UC-OGCs. For further management, our patient was referred to oncology. He was offered neoadjuvant chemotherapy with gemcitabine and paclitaxel, but he preferred to proceed with surgical resection first. He was lost to follow-up.

## Discussion

Pancreatic cancer represents the second most common gastrointestinal malignancy, with adenocarcinoma being the most common subtype [3]. Undifferentiated carcinomas are an exceedingly rare subtype of pancreatic cancer, with an incidence of less than 1% recorded in the literature [3-5]. In 2000, the World Health Organization (WHO) classified undifferentiated pancreatic tumors into two types, undifferentiated carcinoma of the pancreas (UDC) and UC-OGCs [6]. Previously, UC-OGCs had been classified into three different subtypes, namely, osteoclastic, pleomorphic, and mixed; however, in 2010, the WHO grouped them as one entity under the term UC-OGCs [4].

UDC is a highly malignant variant consisting of high mitotic activity with early local and systemic invasion. Histologically, it consists of large eosinophilic pleomorphic cells and ovoid-to-spindle-shaped cells that grow in cohesive formation. Compared to UDC, UC-OGC is composed of both pleomorphic-to-spindle-shaped cells and carries a better prognosis. A mixed subtype has been proposed by Loya et al. and Ezenekwe et al. with features resembling both UDC and UC-OGC [7,8].

From previous reports, UC-OGC tends to occur more commonly in elderly women, with a mean age of presentation of approximately 63 years. The most common clinical presentations of UC-OGC are similar to any other types of pancreatic tumors including upper abdominal pain and weight loss [9]. Our patient presented with epigastric pain and significant weight loss. Other less common clinical manifestations include loss of appetite, steatorrhea, nausea, jaundice, and anemia [10]. Though UC-OGCs of the pancreas are mostly found in the body or tail, our patient was found to have a lesion in the head of the pancreas on a CT scan which was later confirmed with an ultrasound-guided biopsy. The presence of non-neoplastic OGCs on microscopy is the hallmark of UC-OGC [11].

UC-OGC can be either pure or associated with other more common pancreatic tumors such as pancreatic ductal adenocarcinoma and mucinous cystic neoplasm. The origin of OGCs in UC-OGC is not well understood. It was thought to originate from mononuclear histiocytes/macrophages due to their nuclear features along with expression of CD68, vimentin, and lack of reactivity to cytokeratin. Their migration was thought to be due to chemotactic factors produced by the cancerous cells. Areas of necrosis, calcifications, and osteoid bone formation can be observed as well [7]. On immunohistochemical stains, mononuclear neoplastic cells are usually positive for vimentin, keratin, and antibodies to p53. However, OGCs are negative for keratin and p53 antibodies but are positive for CD68, vimentin, and leukocyte common antigen. Our case was confirmed as UC-OGC by typical microscopy appearance, positive CD68, and a 30% proliferation index among neoplastic cells.

Treatment guidelines are limited and most of the information is obtained from isolated case reports/series. Surgery is usually the first line with unpleasant outcomes in most cases due to early recurrence and mortality. The role of radiation and neoadjuvant chemotherapy is extremely limited due to the rarity of the tumor. Limited evidence exists on the use of cisplatin and gemcitabine due to the epithelial origin of the tumor and favorable response. Our patient was referred to higher centers for surgical resection and was subsequently lost to follow-up [12-14].

The prognosis of UC-OGC was found to vary widely with time from diagnosis to death varying from four months to ten years in a study by Togawa et al. [15]. They observed a prolonged survival period associated with surgical resection with an average survival time of 19.6 months (about one and a half years); however, the unoperated group had an average survival time of 6.5 months. Even though not everyone is amenable to surgery, the above findings suggest better survival rates with resection. Other studies have reported poor prognosis among UC-OGC patients with the presence of K-ras oncogene mutations, p53 mutation, and loss of E-cadherin [16,17]. A meta-analysis by Kobayashi et al. comparing short-term and long-term survivors of UC-OGC who underwent surgical resection demonstrated that short-term survivors were noted to be elderly males with smaller tumors and positive lymph node metastasis with a concomitant component of ductal adenocarcinoma.

## Conclusions

Pancreatic osteoclast-like giant cell tumor is an extremely uncommon and complex type of pancreatic cancer with unique characteristics and histopathology. Currently, surgery is the first-line treatment, but the role of radiotherapy and adjuvant/neoadjuvant chemotherapy is not well elucidated. Performing randomized trials is not feasible due to the rarity of the tumor type; hence, maintaining an international registry might help to provide more information to devise potential treatment strategies for this tumor type.

## References

[1] Bray F, Ferlay J, Soerjomataram I, Siegel RL, Torre LA, Jemal A (2018). Global cancer statistics 2018: GLOBOCAN estimates of incidence and mortality worldwide for 36 cancers in 185 countries. CA Cancer J Clin.

[2] Molberg KH, Heffess C, Delgado R, Albores-Saavedra J (1998). Undifferentiated carcinoma with osteoclast-like giant cells of the pancreas and periampullary region. Cancer.

[3] Moore JC, Bentz JS, Hilden K, Adler DG (2010). Osteoclastic and pleomorphic giant cell tumors of the pancreas: a review of clinical, endoscopic, and pathologic features. World J Gastrointest Endosc.

[4] Temesgen WM, Wachtel M, Dissanaike S (2014). Osteoclastic giant cell tumor of the pancreas. Int J Surg Case Rep.

[5] Jo S (2014). Huge undifferentiated carcinoma of the pancreas with osteoclast-like giant cells. World J Gastroenterol.

[6] Klöppel G, Hruban RH, Longnecker DS, Adler G, Kern SE, Partanen TJ (2000). Ductal adenocarcinoma of the pancreas. WHO Classification of Tumors. Pathology and Genetics of Tumors of the Digestive System.

[7] Loya AC, Ratnakar KS, Shastry RA (2004). Combined osteoclastic giant cell and pleomorphic giant cell tumor of the pancreas: a rarity. An immunohistochemical analysis and review of the literature. JOP.

[8] Ezenekwe AM, Collins BT, Ponder TB (2005). Mixed osteoclastic/pleomorphic giant cell tumor of the pancreas: a case report. Acta Cytol.

[9] Guo YL, Ruan LT, Wang QP, Lian J (2018). Undifferentiated carcinoma with osteoclast-like giant cells of pancreas: a case report with review of the computed tomography findings. Medicine (Baltimore).

[10] Rustagi T, Rampurwala M, Rai M, Golioto M (2011). Recurrent acute pancreatitis and persistent hyperamylasemia as a presentation of pancreatic osteoclastic giant cell tumor: an unusual presentation of a rare tumor. Pancreatology.

[11] Gao HQ, Yang YM, Zhuang Y, Liu P (2015). Locally advanced undifferentiated carcinoma with osteoclast-like giant cells of the pancreas. World J Gastroenterol.

[12] Goldberg RD, Michelassi F, Montag AG (1991). Osteoclast-like giant cell tumor of the pancreas: immunophenotypic similarity to giant cell tumor of bone. Hum Pathol.

[13] Joo YE, Heo T, Park CH (2005). A case of osteoclast-like giant cell tumor of the pancreas with ductal adenocarcinoma: histopathological, immunohistochemical, ultrastructural and molecular biological studies. J Korean Med Sci.

[14] Togawa Y, Tonouchi A, Chiku T (2010). A case report of undifferentiated carcinoma with osteoclast-like giant cells of the pancreas and literature review. Clin J Gastroenterol.

[15] Paal E, Thompson LD, Frommelt RA, Przygodzki RM, Heffess CS (2001). A clinicopathologic and immunohistochemical study of 35 anaplastic carcinomas of the pancreas with a review of the literature. Ann Diagn Pathol.

[16] Yonemasu H, Takashima M, Nishiyama KI, Ueki T, Yao T, Tanaka M, Tsuneyoshi M (2001). Phenotypical characteristics of undifferentiated carcinoma of the pancreas: a comparison with pancreatic ductal adenocarcinoma and relevance of E-cadherin, alpha catenin and beta catenin expression. Oncol Rep.

[17] Kobayashi S, Nakano H, Ooike N, Oohashi M, Koizumi S, Otsubo T (2014). Long-term survivor of a resected undifferentiated pancreatic carcinoma with osteoclast-like giant cells who underwent a second curative resection: a case report and review of the literature. Oncol Lett.

